# Evaluating characteristics of PROSPERO records as predictors of eventual publication of non-Cochrane systematic reviews: a meta-epidemiological study protocol

**DOI:** 10.1186/s13643-018-0709-6

**Published:** 2018-03-09

**Authors:** Juan Ruano, Francisco Gómez-García, Jesús Gay-Mimbrera, Macarena Aguilar-Luque, José Luis Fernández-Rueda, Jesús Fernández-Chaichio, Patricia Alcalde-Mellado, Pedro J. Carmona-Fernandez, Juan Luis Sanz-Cabanillas, Isabel Viguera-Guerra, Francisco Franco-García, Manuel Cárdenas-Aranzana, José Luis Hernández Romero, Marcelino Gonzalez-Padilla, Beatriz Isla-Tejera, Antonio Velez Garcia-Nieto

**Affiliations:** 1 Department of Dermatology, Reina Sofía University Hospital, Menendez Pidal Ave, 14004 Córdoba, Spain; 2 IMIBIC/Reina Sofía University Hospital/University of Córdoba, Menendez Pidal Ave, 14004 Córdoba, Spain; 3 Department of Pharmacy, Reina Sofía University Hospital, Menendez Pidal Ave, 14004 Córdoba, Spain; 4 School of Medicine, University of Córdoba, Menendez Pidal Ave, 14004 Córdoba, Spain

**Keywords:** Meta-epidemiology, Deep learning, Predictive models, PROSPERO, Systematic review protocols, Web scraping

## Abstract

**Background:**

Epidemiology and the reporting characteristics of systematic reviews (SRs) and meta-analyses (MAs) are well known. However, no study has analyzed the influence of protocol features on the probability that a study’s results will be finally reported, thereby indirectly assessing the reporting bias of International Prospective Register of Systematic Reviews (PROSPERO) registration records.

**Objective:**

The objective of this study is to explore which factors are associated with a higher probability that results derived from a non-Cochrane PROSPERO registration record for a systematic review will be finally reported as an original article in a scientific journal.

**Methods/design:**

The PROSPERO repository will be web scraped to automatically and iteratively obtain all completed non-Cochrane registration records stored from February 2011 to December 2017. Downloaded records will be screened, and those with less than 90% fulfilled or are duplicated (i.e., those sharing titles and reviewers) will be excluded. Manual and human-supervised automatic methods will be used for data extraction, depending on the data source (fields of PROSPERO registration records, bibliometric databases, etc.). Records will be classified into *published*, *discontinued*, and *abandoned* review subgroups. All articles derived from *published reviews* will be obtained through multiple parallel searches using the full protocol “title” and/or “list reviewers” in MEDLINE/PubMed databases and Google Scholar. Reviewer, author, article, and journal metadata will be obtained using different sources. R and Python programming and analysis languages will be used to describe the datasets; perform text mining, machine learning, and deep learning analyses; and visualize the data. We will report the study according to the recommendations for meta-epidemiological studies adapted from the Preferred Reporting Items for Systematic Reviews and Meta-Analyses (PRISMA) statement for SRs and MAs.

**Discussion:**

This meta-epidemiological study will explore, for the first time, characteristics of PROSPERO records that may be associated with the publication of a completed systematic review. The evidence may help to improve review workflow performance in terms of research topic selection, decision-making regarding team selection, planning relationships with funding sources, implementing literature search strategies, and efficient data extraction and analysis. We expect to make our results, datasets, and R and Python code scripts publicly available during the third quarter of 2018.

## Background

Meta-epidemiological research is designed to evaluate non-clinical aspects of primary and secondary studies, especially their methodological quality [1]. Epidemiology and the reporting characteristics of systematic reviews (SRs) have been previously described by a research group using a cross-sectional random sample of 300 published reviews at two different points in time: in 2004, by Moher et al. [2], and in 2014, by Page et al. [3]. In both cases, several characteristics of the published reviews were analyzed, taking into account the potential differences between Cochrane and non-Cochrane reviews, focus of study (therapeutic, diagnosis, epidemiology, other), self-reported use of Preferred Reporting Items for Systematic Reviews and Meta-Analyses (PRISMA), and differences in SR features between 2004 and 2014. Those authors estimate that more than 8000 SRs are indexed in MEDLINE annually. This type of research may give us a better picture of the overall state of this synthesis of documents, using a representative sample of finally published reviews. Furthermore, some studies have even looked at discrepancies between protocols and the final published reviews [4, 5]. For example, Tricco et al. found that a third of reviews changed or did not specify the primary outcome when the results of International Prospective Register of Systematic Reviews (PROSPERO) registration records were published [6]. These studies highlighted some new issues related to the usefulness of preparing a priori protocols for developing SRs and meta-analyses (MAs) if there are no controls governing the development and reporting of workflow (repository curators, journal editors and reviewers, etc.).

Recently, Tsujimoto et al. have found that 26% of non-Cochrane records registered during the first year after PROSPERO creation remained unpublished [7]. We recently performed a scoping review (data not published) to scrape protocols from the PROSPERO repository by iteratively running a custom Python script from November 25 to December 1, 2017, from RecordID^1^ “00001” to “80000,” due to the lack of a clear pattern for assigning RecordID to every published registration record and PROSPERO’s limitations for massively mining data using query tools on the user’s web interface [8]. As a result, after a rapid curating process, 20,272 full non-Cochrane registration records were obtained (25.3%), of which only 1042 reviews were finally associated with an article communicating results in a scientific journal (5.1%). These numbers are very impressive and make us question whether there is a new and underestimated source of publication bias in the field of SRs and MAs. The magnitude of the problem may be even greater if we consider that by 2015, the majority of the authors of SRs (78%) published in high-impact journals were neglecting to register the protocols they used [9]. Our group has previously demonstrated that certain factors—such as the number of authors with conflicts of interest (COIs), sources of funding, number of authors, and the architecture of author-affiliation networks—may influence the methodological quality of SRs regarding psoriasis [10, 11]. However, no study has analyzed the influence of PROSPERO registration record features on the probability that the results will be finally reported, thereby indirectly assessing the reporting bias of the registration records of SRs and MAs that have completed all PROSPERO stages.

Our hypothesis is that there may be some features related to non-Cochrane registration records that may help predict which results will be finally published in a scientific journal.

In terms of the abovementioned discrepancies between the number of completed registration records at PROSPERO and the low rate of finally published results, we will conduct a meta-epidemiological study with the following objectives:

- To develop and assess different predictive models of results reporting using deep learning strategies based on data and metadata extracted from both published registration records collected in the PROSPERO repository and derived original articles published as SRs or meta-analyses in scientific journals
- To evaluate the influence of discrepancies between the methodological quality of registration records and SRs on the publication bias found in the PROSPERO repository using AMSTAR, AMSTAR-2, and a modified version of AMSTAR (without reporting items, in the case of protocol assessment)

## Methods

### Search strategy

Web scraping will be performed using a custom Python script and Chrome’s Web Scraper website data extraction tool (http://webscraper.io/) to automatically and iteratively extract the raw data of all completed non-Cochrane registration records stored in the PROSPERO repository (https://www.crd.york.ac.uk/prospero/) from February 2011 to December 2017. We will not perform PROSPERO registration record sampling. Rather, our objective is to obtain the entire universe of non-Cochrane PROSPERO registration records—from the first document to the last one registered just before the date of the web scraping—not a representative sample of them. The search specificity for non-Cochrane PROSPERO registration records is based on Python script designed to recognize only the format of these records, which differs from registration records for Cochrane and non-human studies. These cannot be scraped using our script due to the structural differences in PROSPERO forms between them. Thus, the sensitivity and specificity for the web scraping are 100%.

The extracted data will be stored locally as .csv files, where rows will represent protocols and columns protocol sections.

### Eligibility and screening

Registration records with less than 90% of their sections fulfilled or those that are duplicated (i.e., those sharing titles and reviewers) will be dropped from the dataset. Included registration records must have achieved “all completed stages” status (“preliminary searches,” “piloting of the study selection process,” “formal screening of search results against eligibility criteria,” “data extraction,” “risk of bias [quality] assessment,” and “data analysis”). Finally, these *completed* registration records will be classified into three groups: (a) *published reviews*, if at least one publication with results associated with a registration record is available; (b) *discontinued reviews*, if the authors explain why the results have not been finally published; and (c) *abandoned reviews*, if the results were not published and the authors never explained why. An R script will automatically perform the screening process. After that, the results will be subjected to human verification.

### Data extraction

Table 1 displays all the fields that will be extracted from non-Cochrane registration records. All articles derived from *published reviews* will be obtained by multiple parallel searches using the full registration record “title” and/or “list reviewers” in MEDLINE/PubMed databases and Google Scholar. We will only contact authors to request the final reports associated with completed protocols that will be found after bibliographic database searches. Reviewer, author, article, and journal metadata will be obtained using different sources (e.g., SCOPUS, Web of Science, Google Scholar). Table 2 shows the variables that will be included from among reviewer, author, article, and journal metadata. Data from the selected PROSPERO registration records will be automatically extracted using specific regex R syntax. Human verification of the R script process will be done on a sample of records (20%) by two independent researchers. Data and metadata from authors, journals, and articles associated with full PROSPERO registration records will be extracted manually by several researchers using a specifically designed AppSheet form. Given the expected large amount of manual work, the published articles will be assigned to two different teams. Each member of the same team will mine only certain number of variables; thus, the analysis of every group of variables will be performed by two different researchers. Regression analysis will be conducted using the identification of the research member involved in the data extraction as a fixed factor in the mixed effects logistic regression model.

**Table 1:** Variables that will be extracted from non-Cochrane registration records

Protocol section	Variables
Title, objectives	Focus of review
	Common ICD-10 code
	Number of included studies
	Total number of participants
	Type of interventions
	Meta-analysis included
	Network meta-analysis included
	Economic evaluation included
	Use of terms “systematic reviews” or “meta-analysis” in title of abstract
	SR registration (e.g., PROSPERO) mentioned
	SR protocol mentioned
	Reporting guideline (e.g., PRISMA) mentioned
	Cochrane methods used
Eligibility criteria	Based on study design
	Based on publication status
	Based on language of report
Search methods	Number of databases searched
	Years of coverage reported
	Search terms reported
	Trial registry (e.g., ClinicalTrials.gov) searched
	Number of other sources searches
	Other sources searched
Screening, extraction, and risk of bias assessment methods	Screening method
	Data extraction method
	Study risk of bias/quality formally assessed
	Study risk of bias/quality assessment method
	Study risk of bias/quality assessment tool used
	Study risk of bias/quality assessment incorporated into meta-analysis
Included/excluded studies and participants	Review flow reported
	Reason for exclusion of full-text articles reported
	Gray literature (e.g., conference abstracts) included
Outcomes	Number of outcomes stated
	Primary outcome stated
	Type of primary outcome
	Statistical significance of intervention
	Direction of the effect
Statistical methods	Meta-analysis performed
	Meta-analysis model used
	Statistical heterogeneity investigated
	Heterogeneity statistic inappropriately guided choice of meta-analysis model (e.g., random effects model selected if *I*^2^ > 50%) investigated
	Possibility of publication bias discussed/considered in results, discussion, or conclusion
	Subgroup analysis performed
	Sensitivity analysis performed
	Meta-regression performed
	Network meta-analysis performed
Limitations, conclusions, COIs, and funding	GRADE assessment reported in a summary of findings table or text
	Limitations reported
	Study risk of bias/quality/limitations incorporated into therapeutic SR abstract conclusions
	COIs reported
	Source of funding of the SR

**Table 2:** Variables that will be included into the dataset from reviewer, author, article, and journal metadata

	Variable	Source
Reviewers	H-index	Web of Science
	Institution	PROSPERO protocol
	Country	PROSPERO protocol
	Number of authored publications	SCOPUS, Google Scholar
	Number of authored systematic reviews	SCOPUS, Google Scholar
Authors	H-index	Web of Science
	Institution	Article, Web of Science
	Country of corresponding author	Article, Web of Science
	Number of authored publications	SCOPUS, Google Scholar
	Number of authored systematic reviews	SCOPUS, Google Scholar
Article	Journal name	Web of Science
	Year of publication	Article
	Number of authors	Article
	Number of institutions	Article
	Conflict of interests (COIs)	Article
	Sources of funding	Article
	Dataset and code availability	Article
Journal	Journal impact factor	Web of Science
	Journal type (general, specialty)	–
	Journal rank (quartile)	SCImago
	Peer review quality and transparency of the peer review process	[13]
	Open access vs traditional (subscription) journals	Directory of Open Access Journals (DOAJ)
	PRISMA endorsement	http://prisma-statement.org/Endorsement/PRISMAEndorsers.aspx

### Data analysis and reporting

Qualitative variables will be summarized by level frequency (number, %) or displayed using several types of graphs (mosaic plots, density/histogram plots, etc.). Quantitative variables will be summarized using the mean (standard deviation) or median (interquartile range) for non-normally distributed variables. We will look for features of PROSPERO registration records (Table 1) and metadata (Table 2) that can predict publication (or non-publication) using mixed effects logistic regression models. Results will be displayed using odds ratios (OR). The 95% confidence intervals will be estimated by simple random sampling with replacement for bootstrapping for every variable included in the model.

We plan to look for publications in a random 10% sample of abandoned PROSPERO records following the same method used for the completed and published PROSPERO records. By doing this, we can check the robustness of our approach as well as the ratio of return information by authors after results are published.

R and Python programming and analysis languages will be used to describe the datasets; perform text mining, machine learning, and deep learning analyses; and visualize the data. We will implement actions aimed at making every effort to improve the transparency, reproducibility, and efficiency of our work. First, by publishing this a priori protocol of our meta-epidemiological study, we will avoid publication bias and issues related to analytical flexibility (outcome switching and P-hacking). Second, our raw datasets and R and Python code scripts will be made publicly available in the GitHub repository, using version control and R packages such as *R Markdown*, *knitR*, and *packrat*. Third, we will report the study according to a previously adapted recommendation of the Preferred Reporting Items for Systematic Reviews and Meta-Analyses (PRISMA) statement for meta-epidemiological studies, proposed in 2017 by Murad and Wang [12]. Those authors adapted the items used in the PRISMA statement for systematic reviews and meta-analyses to improve the transparency of this type of research. The items were categorized according to their title (1), abstract (1), introduction (2), methods (10), results (6), discussion (3), and funding (1).

## Discussion

This meta-epidemiological study will explore for the first time, using a large sample of studies, the factors that may be associated with the success of an a priori systematic review registration record, defined as one entailing a high probability that a study’s results will finally be published as an original article in a scientific journal. The findings of this study will be useful in the future for improving protocol design when researchers using non-Cochrane protocols decide to develop a systematic review and meta-analysis. The evidence may also help to improve the performance of review workflow, including selecting a better topic of research, making better decisions regarding team selection, planning relationships with research funding sources, implementing literature search strategies, and performing more efficient data extraction and analysis. Our results, datasets, and R and Python code scripts are expected to be made publicly available during the third quarter of 2018.

## Abbreviations

AMSTAR: A Measurement Tool to Assess the Methodological Quality of Systematic Reviews
MAs: Meta-analyses
PRISMA: Preferred Reporting Items for Systematic Reviews and Meta-Analyses
SRs: Systematic reviews

## Footnotes

1 By replacing XXXXX with the RecordID at https://www.crd.york.ac.uk/prospero/display_record.php?RecordID=XXXXX.
